# Lateral release associated with MPFL reconstruction in patients with acute patellar dislocation

**DOI:** 10.1186/s12891-022-05013-5

**Published:** 2022-02-11

**Authors:** Nadim Kheir, Giuseppe Salvatore, Alessandra Berton, Alexander Orsi, Jonathan Egan, Amin Mohamadi, Joseph P. DeAngelis, Arun J. Ramappa, Umile Giuseppe Longo, Vincenzo Denaro, Ara Nazarian

**Affiliations:** 1 Musculoskeletal Translational Innovation Initiative, Carl J. Shapiro Department of Orthopaedic Surgery, Beth Israel Deaconess Medical Center, Harvard Medical School, Boston, MA USA; 2 Department of Orthopaedic and Trauma Surgery, Campus Bio-Medico University, Rome, Italy; 3 Clinical Research, Corin, Raynham, MA USA; 4 Carl J. Shapiro Department of Orthopaedic Surgery, Beth Israel Deaconess Medical Center, Harvard Medical School, Boston, MA USA; 5 Department of Orthopaedic Surgery, Yerevan State Medical University, Yerevan, Armenia

## Abstract

**Objective:**

Medial patellofemoral ligament (MPFL) injury occurs in the majority of the cases of acute patellar dislocation. The role of concomitant lateral retinaculum release with MPFL reconstruction is not clearly understood. Even though the lateral retinaculum plays a role in both medial and lateral patellofemoral joint stability in MPFL intact knees, studies have shown mixed clinical outcomes following its release during MPFL reconstruction surgery. Better understanding of the biomechanical effects of the release of the lateral retinaculum during MPFL reconstruction is warranted. We hypothesize that performing a lateral release concurrent with MPFL reconstruction will disrupt the patellofemoral joint biomechanics and result in lateral patellar instability.

**Methods:**

A previously developed and validated finite element (FE) model of the patellofemoral joint was used to understand the effect of lateral retinaculum release following MPFL reconstruction. Contact pressure (CP), contact area (CA) and lateral patellar displacement were recorded. abstract.

**Results:**

FE modeling and analysis demonstrated that lateral retinacular release following MPFL reconstruction with tibial tuberosity-tibial groove distance (TT-TG) of 12 mm resulted in a 39% decrease in CP, 44% decrease in CA and a 20% increase in lateral patellar displacement when compared to a knee with an intact MPFL. In addition, there was a 45% decrease in CP, 44% decrease in CA and a 21% increase in lateral displacement when compared to a knee that only had an MPFL reconstruction.

**Conclusion:**

This FE-based analysis exhibits that concomitant lateral retinaculum release with MPFL reconstruction results in decreased PF CA, CP and increased lateral patellar displacement with increased knee flexion, which may increase the risk of patellar instability.

## Introduction

Acute patellar dislocations primarily occur in active young patients [1], with a recurrence rate of 17% after the first dislocation episode and up to 49% after recurrent dislocations [2]. Injury to the medial patellofemoral ligament (MPFL) can be seen in the majority of cases after initial patellar dislocation [3–7]. MPFL is responsible for up to 60% of the restraining force against lateral patellar dislocation [8]. Thus, many authors have advocated for acute treatment of the medial patellofemoral ligament (MPFL) after a first-time patellar dislocation [9–11]. Depending on the extent of injury of the MPFL, primary repair or reconstruction of the MPFL is advocated. In most cases where MPFL reconstruction is warranted, lateral retinacular release is performed at the time of surgery [6, 9, 11–14]. The lateral retinaculum is a complex structure composed of various fascial layers on the anterolateral aspect of the joint [15]. The lateral retinaculum has been proven to affect both medial and lateral patellofemoral joint stability in knees with an intact MPFL [16]. Biomechanical studies on isolated lateral release drive to the conclusion that it should not be performed as a primary treatment for patellofemoral instability [16]. Nevertheless, there are no clear indications about lateral release in combination with acute MPFL reconstruction. The literature points to a scant of amount of comparative prospective studies comparing MPFL reconstruction with and without lateral release. However, when it comes to primary repair of the MPFL, a few clinical studies have shown increased recurrence rates of patellar instability when lateral release is added [12, 17] and only one *in-vitro* study conducted by Bedi et al. investigated the effect of lateral release on patellar stability when an MPFL repair is performed [18]. Bedi et al. measured the force required to displace the patella 1 cm laterally in eight fresh-frozen human cadaveric knees when the MPFL was transected, upon repair, and then when lateral release was added. They noticed that lateral release reduced the required force by 7% to 11% compared with the MPFL-repaired knee. Their findings support the hypothesis that lateral release cannot be routinely performed in primary repair of the MPFL ligament for acute patellar instability. Thus, the role of lateral retinacular release in MPFL reconstruction surgery as the treatment for patellar instability should be investigated.

The aim of this study is to study the effect of lateral release after acute MPFL reconstruction using a previously validated patellofemoral finite element model [19]. As the lateral retinaculum has been shown to contribute to medial and lateral stabilization of the patella in knees with an intact MPFL [16], *we hypothesize that performing a lateral release when performing MPFL reconstruction will alter the biomechanics of the patellofemoral joint and increase the lateral instability of patella.* Understanding the biomechanics of this procedure will further help elucidate the best surgical approach for acute patellar dislocations.

## Methods

### Conditions

**Fig. 1:**
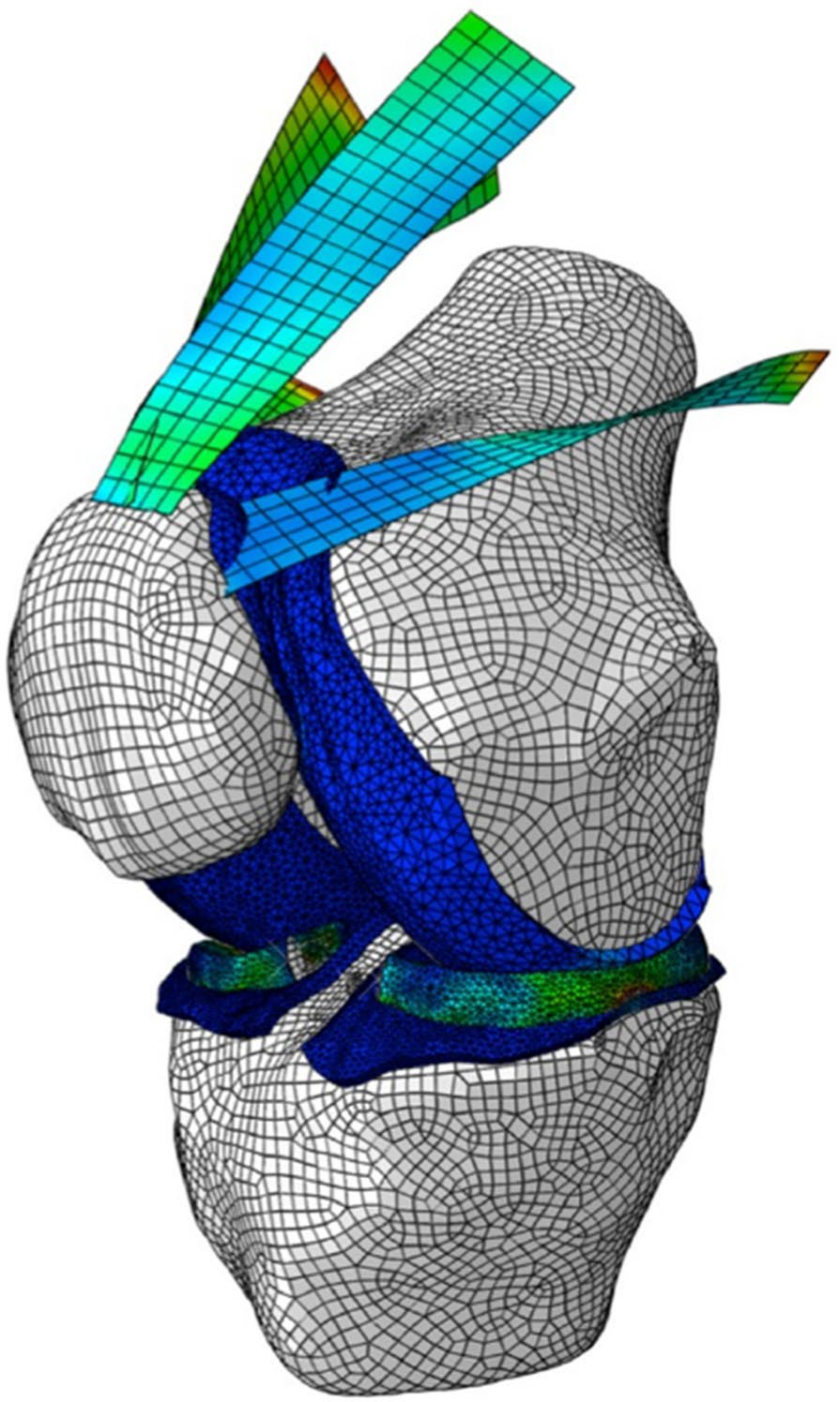
FE model of the knee joint. It shows the patellofemoral joint (PFJ) articular cartilage, medial patellofemoral ligament (MPFL), and the quadriceps muscles

A previously developed and validated finite element (FE) model of the patellofemoral joint (PFJ) was used to simulate the conditions to understand the effect of lateral retinaculum release following MPFL reconstruction [19]. The FE mesh model is illustrated in Fig. 1. According to previous biomechanical studies, the following parameters were used in the FE model: a total load of 175 N was applied to the quadriceps muscle group, a load of 30 N to the iliotibial band, a stiffness of 49 N/mm for the patellar tendon, a stiffness of 16 N/mm for the MPFL and a stiffness of 97 N/mm for the lateral retinaculum [20–23]. The quadriceps muscle was divided into five components represented by 2D quadrilateral mesh elements. The direction and tension of each component were taken from literature [16, 24]. Contact interactions between cartilage and cartilage, and cartilage and menisci were simulated using a frictionless finite-sliding formulation [25, 26].

The transepicondylar axis was used as axis of rotation to simulate knee flexion [27]. Femoral degrees-of-freedom and tibial medial–lateral translation and internal–external rotation were constrained; the remaining tibial degrees-of-freedom were left free. The patella was unconstrained for all six free degrees-of-freedom.

In addition to the healthy, intact condition with a TT-TG of 12 mm, we created 3 variations of the model to sequentially examine patellar instability, MPFL reconstruction, and lateral release (Table 1).

**Table 1:** Characteristics of investigated conditions in the FE model

Conditions	TT-TG (mm)	MPFL	Lateral Retinaculum
*Healthy*	12	MPFL stiffness/Intact	Native stiffness/Intact
*Patellar Instability*	12	Deactivated/Transected	Native stiffness/Intact
*MPFL Reconstruction*	12	Semitendinosus stiffness/Reconstructed	Native stiffness/Intact
*MPFL Reconstruction* + *Lateral Resection*	12	Semitendinosus stiffness/Reconstructed	Deactivated/Transected

The intact model was altered to simulate injury: spring elements representing MPFL were deactivated, since the MPFL is injured in the majority of patients after dislocation [3–7]. This injured model was then modified to simulate MPFL reconstruction: the MPFL spring elements were reactivated, but the stiffness was set to 20 N/mm which corresponds to the stiffness of the semitendinosus tendon (i.e., the tendon used for reconstruction) [28]. Finally, the reconstruction model was changed by deactivating the spring elements representing the lateral retinaculum in order to simulate lateral release. Contact pressure (CP) and contact area (CA) were recorded between the patella and the trochlear groove. The patellar kinematic (lateral displacement) were recorded relative to a standard reference of the intact knee at 0° of flexion. PFJ CP, CA and lateral displacement were measured at 0°, 5°, 10°, 15°, 30°, 60°, and 90° of knee flexion for each tested condition.

## Results

### Contact Pressure

**Fig. 2:**
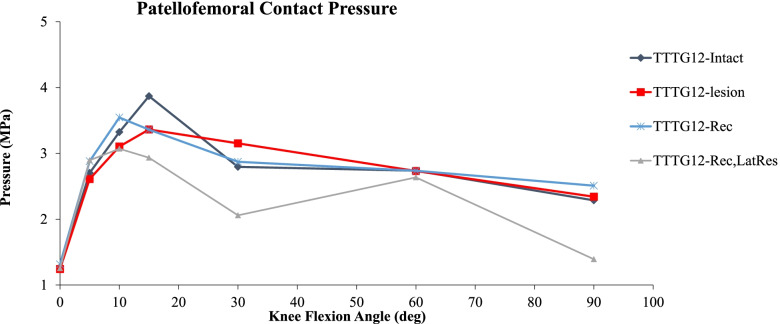
Comparison of PF CP FE simulations between: TT-TG 12 Baseline condition (purple diamond), patellar instability group: TT-TG 12 and MPFL injured (red square), reconstruction group: TT-TG 12 and MPFL reconstruction (blue star), and reconstruction group with concomitant lateral release (pink triangle). Mean contact pressures (MPa) are illustrated across knee flexion angles of 0 degrees (full extension) to 90 degrees of flexion

Patellofemoral CPs of the different conditions approximated by the FE model are illustrated in Fig. 2. The injured condition shows an 11% increase in contact pressure compared to the intact model at 30° knee flexion angle before having equal contact pressure at 60°. The reconstruction of the MPFL most closely approximates the intact condition, with a 13% decrease in CP only at 15°. Performing a lateral release, following an MPFL reconstruction, results in lower CP throughout knee flexion with a difference of 27% at 30° and 39% at 90° of knee flexion respectively compared to the intact model. These differences are similar when comparing MPFL reconstruction with and without lateral release, which results in a 28% decrease of CP at 30° and 45% at 90° of knee flexion in the knee with lateral release. Interestingly at 60° of knee flexion, the CP of the MPFL reconstruction with lateral release condition approximates that of the other conditions with only 4% difference.

### Contact Area

**Fig. 3:**
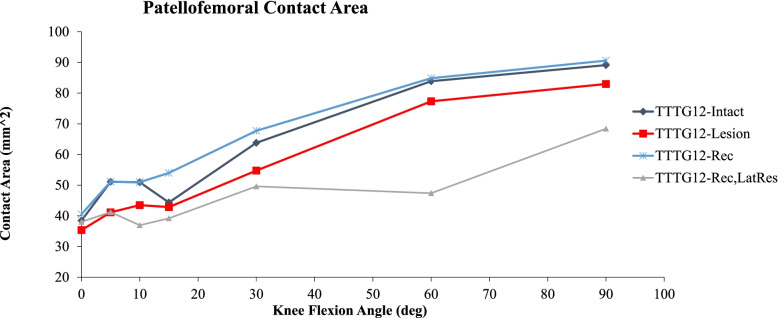
Comparison of PF CA FE simulations between: TT-TG 12 Baseline condition (purple diamond), patellar instability group: TT-TG 12 and MPFL injured (red square), reconstruction group: TT-TG 12 and MPFL reconstruction (blue star), and reconstruction group with concomitant lateral release (pink triangle). Mean CA (MPa) are illustrated across knee flexion angles of 0 degrees (full extension) to 90 degrees of flexion

Patellofemoral CAs of the different approximated by the FE model are illustrated in Fig. 3. The injured condition shows a reduced CA at all knee flexion angles with a maximum difference of 15% to the intact condition and a minimum of 3% difference only at 30° of knee flexion. Reconstruction of the MPFL shows only a 1% increase in CA compared to the intact condition, with an 18% increase only at 15°. Performing a lateral release, following an MPFL reconstruction, results in a minimum decrease of 12% of CA at 15° and a maximum of 44% decrease of CA at 60° when compared to the intact condition. In addition, the MPFL reconstruction with lateral release showed a decrease of 27% of CA at 15° and a 44% decrease of CA at 60° when compared with condition with only an MPFL reconstruction.

### Lateral Patellar Displacement

**Fig. 4:**
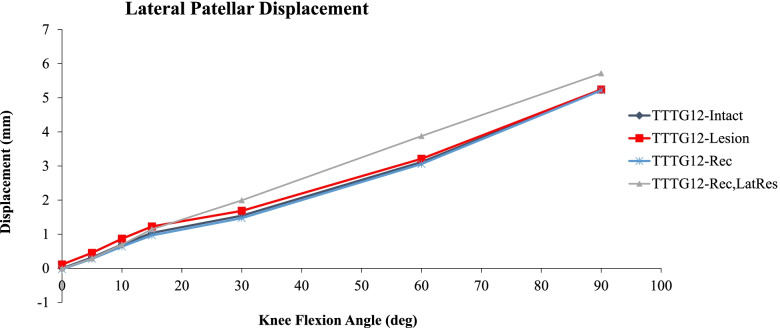
Comparison of lateral patellar displacement FE simulations between: TT-TG 12 Baseline condition (purple diamond), patellar instability group: TT-TG 12 and MPFL injured (red square), reconstruction group: TT-TG 12 and MPFL reconstruction (blue star), and reconstruction group with concomitant lateral release (pink triangle). Mean lateral patellar displacement (mm) are illustrated across knee flexion angles of 0 degrees (full extension) to 90 degrees of flexion

Lateral patellar displacement of the different conditions approximated by the FE model are illustrated in Fig. 4. At knee flexion angles less than 15°, the lesion model increased the lateral patellar displacement by 20% compared to the rest of the conditions. Lateral release with MPFL reconstruction showed increased lateral patellar displacement compared to the other conditions starting at 20° reaching a maximum increase of 21% at 60° of knee flexion angle.

## Discussion

The results from the FE model show that lateral retinacular release following MPFL reconstruction in knees with TT-TG of 12 mm have decreased CP, CA and increased lateral patellar displacement with increasing knee flexion. With decreasing CP and CA, lateral release offloads the patella in the patellofemoral joint, which is why it is advocated in patients suffering from lateral patellar compression syndrome [29]. However, the decrease in PF contact and increase in lateral patellar displacement following lateral release (LR) may increase the risk of patellar instability. This can explain the lesser amount of force needed to displace the patella laterally in the study by Bedi et al. [18]. In addition, using the same FE model, these results are in line with the results when lateral release is performed concomitantly with a tibial tuberosity osteotomy to treat patellar instability when the TT-TG distance is greater than 12 mm [19]. Thus, regardless of the procedure being performed for patellar instability, the addition of LR will decrease the patellofemoral joint CA, CP and increase the lateral patellar displacement.

Clear indications for the concomitant release of the lateral retinaculum during MPFL reconstruction surgery do not exist [30]. A systematic review done by Migliorini et al showed that some surgeons advocate concomitant release of the lateral retinaculum in all patients undergoing MPFL reconstructions, while others had various criteria on when it should be performed [30]. In the systematic review, it showed that patients that had lateral release had higher patient reported outcome measures (PROMs) compared to those that did not get a lateral release, but the results were not statistically significant [30]. These results are in parallel with Malatray et al that showed a non-statistically significant higher functional score in the lateral release group [31]. Migliorini et al hypothesized that the unloading mechanism of the lateral release on the lateral patellar compartment, which is injured in the majority of acute patellar dislocations [32], may be the contributing factor to the increased PROMs in patients with LRR [30]. As seen by the FE model, when a lateral release is performed during MPFL reconstruction, the CA and CP between the patella and trochlear groove decreases with increasing knee flexion. This decreased CA and CP confirm the hypothesis of Migliorini et al and can explain the increased PROMs in the patients that had a lateral release. However, this unloading mechanism of the lateral release has no effect on the development or progression of osteoarthritis, as outlined by Nomura et al, where after a mean follow-up of 11.9 years, osteoarthritis between patients that had an MPFL with and without a lateral release were the same [33].

The criteria for concomitant release of the lateral retinaculum during MPFL reconstruction surgery is heterogenous in nature and based on surgeon preference [30]. Surgeons base their indication upon examination of the tension of the lateral structures and their effects on patellar maltracking post-MPFL reconstruction [30]. Han et al., on the other hand, advocates the use of lateral release in all patients undergoing MPFL reconstruction, stating that it improves post-operative range of motion [34]. In the FE model, it was shown that lateral release will cause increased lateral displacement in increasing knee flexions. Ultimately, this will lead to the conclusion of increased risk of lateral patellar instability. However, in the systematic review by Migliorini, the positive apprehension test and rates of dislocations amongst patients that had a lateral release and those that did not were similar [30]. In addition, in a prospective study conducted by Wang et al., where patients with MPFL reconstruction with and without LR were followed up for 1 year post-operatively, reported that the LR group had significantly better functional outcome scores and did not increase the risk of complications [35]. Thus, precise criteria for the use of lateral release need to be outlined in order to assist surgeons in the approach of the management of patients suffering from lateral patellar dislocation.

### Limitations

The limitations of the study are related to the use of the FE model in stimulating the conditions needed to understand the role of lateral release in knees with MPFL reconstruction. The model used is based on a normal knee of which different conditions were applied on and it does not take into account other risk factors for patellar instability that include patella alta, trochlear dysplasia, and lateralization of the tibia tubercle. In addition, the FE model did not present patellar tilt. Not all patients with patellofemoral instability have patellar tilt, but for those with significantly abnormal patellar tilt on preoperative examination and radiographic studies, lateral release may be required for the patella to track normally. Finally, to reproduce MPFL reconstruction in this model, reactivation of the MPFL spring elements with use of the stiffness of the semitendinosus was done. However, variations in MPFL reconstruction surgery, which include the type of graft, number of bundles, and amount of graft tensioning, play a role in the outcome of the surgery of which the FE model does not take into account for.

## Conclusion

In conclusion, this FE model showed that concomitant lateral retinaculum release with MPFL reconstruction results in decreased PF CA, CP and increased lateral patellar displacement with increased knee flexion, which may increase the risk of lateral patellar instability. Surgeons need to understand the biomechanical effects of lateral release on the PFJ during MPFL reconstruction and use it cautiously when indicated.

## Data Availability

The data-sets used and/or analysed during the current study available from the corresponding author on reasonable request.
